# Concurrent use of herbal and prescribed medicine by patients in primary health care clinics, South Africa

**DOI:** 10.4102/phcfm.v15i1.3829

**Published:** 2023-06-29

**Authors:** Tebogo Tsele-Tebakang, Heather Morris-Eyton, Erica Pretorius

**Affiliations:** 1 Department of Complementary Medicine, Faculty of Health Sciences, University of Johannesburg, Johannesburg, South Africa; 2 Department of Sport and Movement Studies, Faculty of Health Sciences, University of Johannesburg, Johannesburg, South Africa; 3 Centre for Academic Technologies, Academic Development and Support, Faculty of Health Sciences, University of Johannesburg, Johannesburg, South Africa

**Keywords:** herbal medicine (HM), primary health care providers, primary health care nurses, herb-drug interaction, Primary health care clinics, African traditional medicine (ATM)

## Abstract

**Background:**

The use of herbal medicine (HM) as a self-management practice for treating various diseases has gained popularity worldwide. Consumers co-administer herbal products with conventional medicine without the knowledge of possible herb-drug interaction (HDI).

**Aim:**

This study aimed to assess patients’ perception and use of HM and their knowledge of HDI.

**Setting:**

Participants attending primary health care (PHC) clinics in three provinces (Gauteng, Mpumalanga and Free State), South Africa, were recruited.

**Methods:**

Focus group discussions comprising a total of thirty (*N* = 30) participants were conducted using a semi-structured interview guide. Discussions were audio-recorded and then transcribed verbatim. Data were analysed using thematic content analysis.

**Results:**

Reasons for using HM, sources of information on HM, co-administration of HM and prescribed medicine, disclosure of the use of HM, PHC nurses’ attitudes and not having time to engage were frequently discussed. Respondents’ lack of knowledge and perceptions about HDI and their dissatisfaction with prescribed medicine because of experienced side effects were also discussed.

**Conclusion:**

Because of the lack of discussions and non-disclosure about HM in PHC clinics, patients are at risk of experiencing HDIs. Primary health care providers should regularly enquire about HM use on every patient, to identify and prevent HDIs. The lack of knowledge about HDIs by patients further compromises the safety of HM.

**Contribution:**

The results highlighted the lack of knowledge of HDI by patients thus assisting the healthcare stakeholders in South Africa to implement measures to educate patients attending PHC clinics.

## Introduction

People worldwide have been using medicinal herbs to improve or treat their health conditions before the inception of conventional medicine. Over time, the world has seen an increased demand for herbal products.^1^ African traditional medicine (ATM) particularly herbal medicine (HM) has been part of health care in African countries, and its contributions to the primary health care (PHC) system are well known in some parts of the African continent.^2^ South Africa categorises indigenous HM as ATM, whereas Homeopathy, Ayurveda, Traditional Chinese Medicine, Unani Tibb, and Western Herbal Medicine, as complementary medicine (CM).^3^ However, these modalities are not acknowledged as part of the national healthcare system.^4^ South Africa, which is a country rich in indigenous medicinal plants, has approximately 30 000 plant species, accounting for almost 10% of the world’s higher plant species.^5^ A large proportion (60%–80%) of the South African population uses herbal products for the treatment of ailments and cultural practices.^6^

These practices are derived from the indigenous knowledge and experiences possessed by communities. Indigenous knowledge (IK) is regarded as the knowledge that has been acquired outside the educational system. It is easily transferred in a community that shares common cultural concepts and who speaks the same language.^7^ Indigenous knowledge is central to the social, cultural and spiritual beliefs of these communities.^8^ The deep-rooted indigenous knowledge of HM is evident in the decision-making and usage of HM.^9^ Moreover, a personal, family and community-positive experience, easeof availability, perceived lack of side effects and patients’ experience or witness of unfavourable behaviour of healthcare providers (HCPs) is a push factor towards using HM.^10^

Herbal products can either be obtained from a traditional health practitioner (THP), herbal markets or pharmacies as well as being available as home-made remedies.^11^ South Africa has taken various initiatives to promote the safety, efficacy, labelling and traditional use of HM.^12^ However, most of these are not registered by the South African Health Products Regulatory Authority (SAHPRA) and have not undergone quality control tests and their safety is unknown.^13,14^ According to the World Health Organization (WHO), HM comprised herbs, herbal materials and preparations and finished products that contain plants as active constituents.^15^ These herbs may contain more than 150 active constituents that may affect the pharmacokinetics of prescribed medicines and cause adverse effects.^16^ These adverse effects range from an allergic reaction, gastrointestinal upset, coagulation abnormalities, cerebral haemorrhage, liver damage and possible death.^17,18^

In South Africa, herbal products are sold at herbal markets or by THP as herbal concoctions, packaged in recyclable plastic bottles or paper with labels that do not provide the full ingredients of the herb.^19^ These herbal concoctions are a mixture of different plant species or a single plant to treat various health ailments.^20^ Patients use these herbal concoctions concurrently with prescribed medicines without the knowledge of the implications of HDIs.^21,22^ Research shows that desperate patients use HM to complement prescribed medicines such as antihypertensive, hypoglycaemic, antiarrhythmic, anticoagulant, antiretroviral and antidepressants.^12,16^ Moreover, a review conducted to assess the severity of HDIs in patients who co-administer HM and prescribed medicines showed that patients taking warfarin, insulin, aspirin and/or statins, reported evident interactions with herbal products including: (1) sage, (2) flaxseed, (3) cranberry, (4) *Camelia sinesis,* (5) *Hypericum perforatum*, (6) *Ginkgo biloba* and (7) *Matricaria chamomile*.^23,24^ For example, acetylsalicylic acid as an antiplatelet agent is the most drug that interacts with HMs, followed by warfarin that can interact with *Ginkgo biloba* and *Hypericum perforatum,* leading to increased bleeding.^25^ Amlodipine an antihypertensive drug when combined with *Ginkgo biloba* changes the pharmacokinetic interactions of amlodipine through the metabolism of CYP3A4 enzyme.^26^

Several studies conducted in South Africa confirmed that consumers do not disclose the use of HM to their healthcare providers.^27,28,29^ The non-disclosure prevents the discussion and recording of potential HDIs by HCPs.^30^ Additionally, the non-registration of most HM exacerbates the risk of HDIs.^14^ Several studies have explored the reasons why patients use HM^31,32,33^, but within the South African context, there is a dearth of information regarding patients’ knowledge about herb-drug interaction.

This study sought to provide an in-depth description of the use of HM through focus group discussions (FGD) with patients attending PHC clinics in three provinces – Gauteng, Free State and Mpumalanga, South Africa.

## Research methods and design

### Research design and setting

This study used a descriptive qualitative method to collect data using focus groups from three provinces in South Africa, namely: Gauteng, Mpumalanga and Free State.

### Research population and sampling strategy

The study targeted participants 18 years and above who had similar experiences in attending PHC clinics. Purposive sampling was employed, where participants acknowledged co-administration of HM and prescribed medicines. Focus group discussions were held in each province and comprised 10 participants. The FGDs were conducted in local community churches, a place that is familiar to the participants, allowing them to have the freedom to provide information that can be difficult to express in primary health care facilities.^34^

### Data collection

Data were collected using audio-recorded, semi-structured discussions that lasted between 60 min and 120 min. To recruit participants, the researcher approached participants from the PHC clinics, the community and local churches in person where the purpose of the study was explained and a request to participate was made. Participants were informed that they are free to withdraw from the study at any point of the discussion without prejudice. Participants who agreed to participate in the study were requested to sign the consent forms to participate as well as for audio recording prior to data collection. The recruitment procedure followed was similar in all provinces. Three focus groups were sufficient to identify a variety of new insights to reach saturation.^35^ The discussions were conducted in isiZulu and Sesotho, which are the main languages spoken by participants, then translated and transcribed into English. A research assistant who is a master’s student and able to speak the languages was present in all FGD to take notes on non-verbal expressions that could not be audio-recorded. A pilot study was utilised to test the guide for ease of understanding. The researcher who received training in facilitating FGDs used her communication skills to encourage participants to volunteer information freely.^36^ The discussions were standardised by using the same interview guide in all provinces where each discussion began with the following question: ‘What do you understand about herbal medicine?’. Further questions included:

- Which herbal products do you use?
- Why did you choose to use herbal medicine?
- Do you use herbal products together with conventional medicine?
- Do you tell the concurrent use of herbal medicine and conventional medicine to your healthcare providers?
- If not, why not?
- If yes, what was the response?
- Tell me what you understand by herb-drug interaction?
- What do you think can encourage communication about herbal medicine use between patients and healthcare providers?
- Any comments?

Triangulation was employed to collect and analyse the data, to enable the convergence of diverse experiences from three provinces and to enhance a better understanding of the findings.

### Data analysis

The translated transcripts were verified by the co-coder, where themes developed around the topic of the concurrent use of herbal and prescribed medicine and potential HDI. Thematic content analysis was conducted to extrapolate data from transcripts using Atlas ti version 9 software.

### Ethical considerations

The research was approved by University of Johannesburg, Research Ethics Committee (REC-01-106-2018) prior to conducting the research.

### Trustworthiness of the data

Trustworthiness of the findings was ensured throughout this study as described by Lincoln and Guba, 1985 in Polit & Beck.^37^ *Credibility* was promoted by recorded, in-depth discussions where participants voluntarily agreed to participate in the study. *Transferability* was ensured by providing research methodology and a description of the findings to enhance the transparency of the study. To determine *reliability*, the researcher made sure that replication of this study is possible and, that similar findings can be established. To ensure *confirmation*, the researcher can provide an audit trail of the analysis and findings of the study.

## Results

### Participant’s characteristics

Thirty individuals participated in this study. Females, 29 (96.7%), and a male, 1 (3.3%). The median age was 48 years. The statistics showed that most respondents, 22 (73.3%) spoke Sesotho and 8 (26.7%) spoke isiZulu (Table 1). To ensure anonymity, every quote is reported according to the focus group number, gender and the language spoken. Example: FG1, F, IsiZulu. Based on the results, thematic analysis revealed five themes and three sub-themes (Table 2).

**TABLE 1:** Demographics data of respondents.

Variables	Frequency	%
**Age(years)**		
18–30	8	26.7
31–40	2	6.7
41–50	1	3.3
51–60	9	30
60+	10	33.3
**Gender**		
Male	1	3.3
Female	29	96.7
**Language spoken**		
IsiZulu	8	26.7
Sesotho	22	73.3

**TABLE 2:** Themes and sub-themes on the concurrent use of herbal medicine and prescribed medicine by patients.

Themes	Sub-theme
1. Reasons for using herbal medicine	Dissatisfaction with conventional medicine
2. Source of information on herbal medicine	-
3. Co-administration of herbal medicine with conventional medicine	Knowledge and perceptions about herb-drug interaction
4. Disclosure of the use of herbal medicine	-
5. Healthcare providers do not have time to engage	Nurses’ attitude

### Theme 1: Reasons for using herbal medicine

Respondents mentioned that HM is cheaper, easily accessible, has fewer side effects and they have been using HM since childhood:

‘Because herbs are based on natural plants and have been used throughout ancient days by our ancestors to cure ailments before the discovery of doctors’ medicines.’ (FG3, F, isiZulu)

‘Herbs are less expensive than doctors’ medicine.’ (FG3, F, isiZulu)

‘They are cheaper.’ (FG2, F, Sesotho)

‘As black people, we grew up believing that this is exactly what you need for a particular problem.’ (FG2, F, Sesotho)

‘They have less side-effects.’ (FG1, F, Sesotho)

‘Easy access. You don’t have to go through doctors.’ (FG2, F, Sesotho)

‘It’s easy to take this one because it’s always there at home, the doctor is far away.’ (FG2, F, Sesotho)

#### Sub-theme: Dissatisfaction with conventional medicine

Respondents reported their disappointment with conventional medicine. Their main worry was the side effects of conventional medicines:

‘Yes, we just take the medicine as it is and be surprised by side effects when we get home.’ (FG3, F, isiZulu)

‘They once dispensed pills that made had strong side effects without explaining it to me. I stopped taking the medication on my own.’ (FG2, F, Sesotho)

‘Because of the side effects that conventional medicine gives us, we end up not drinking the right dose because we are scared that it can harm us.’ (FG1, F, Sesotho)

### Theme 2: Source of information on herbal medicine

Respondents reported mostly hearing about herbal medicine from word of mouth:

‘Sometimes when we are sick or not well, we take advice from people in our communities about herbs because we need help to remedy the illness.’ (FG3, F, isiZulu)

‘We hear from the TV or word of mouth about herbs then we end up buying. And we don’t need to consult the doctor as it is readily available for us.’ (FG1, M, Sesotho)

‘Mostly because of word of mouth, when you hear that herbs have healed someone you end up buying them also.’ (FG1, M, Sesotho)

‘Word of mouth. Another one will say: ‘“Take this one, it works.”’ (FG2, F, Sesotho)

### Theme 3: Co-administration of herbal medicine with prescription medicine

Respondents mentioned that they mix herbal medicine and clinic or doctors’ medicines:

‘We are used to mixing all the medication same time. The medication that the doctor gives us and the one we buy from the pharmacy.’ (FG1 F, Sesotho)

‘I think another thing is you wouldn’t think it has danger in it. Like you think if it’s medication, I am going to get better. If I take it together, more effective in a way.’ (FG2 F, Sesotho)

‘We are mixing them.’ (FG3, F, Sesotho)

However, there were a few respondents who mentioned that they do not mix herbal medicine and prescribed medicines:

‘As much as I am not educated, by God’s wisdom I could sense that is not right to mix the medication.’ (FG1, F, Sesotho)

#### Sub-theme 2: Knowledge about herb-drug interaction

Most of the respondents did not know about herb-drug interaction, and that it was harmful to take these medicines at the same time, while some had a feeling that it was dangerous to mix them:

‘No, I don’t know.’ (FG1, F, Sesotho; FG2, F, Sesotho and FG3, F, isiZulu)

‘No, I did not know about that but the nurse at the clinic advises us not to mix them but we can continue to take the herbs.’ (FG1, F, Sesotho)

‘No, I did not know. When I bought them the Dr said I can take them with clinic pills there is no problem.’ (FG3, F, isiZulu)

‘Yes, I can see that they can harm you if you mix them.’ (FG2, F, Sesotho)

‘Yaa, I am not taking them at the same time. I just thought to myself it will not be ok because I have already taken the other ones.’ (FG2, F, Sesotho)

### Theme 4: Disclosure of the use of herbal medicine

Respondents mentioned that they do not disclose the use of herbal medicine to their healthcare providers:

‘I do not tell them.’ (FG3, F, isiZulu)

‘I didn’t tell the doctor about the immune boosters, I just pretended like I don’t know the cause of my high blood pressure.’ (FG1, F, Sesotho)

‘The thing is we don’t tell them the whole truth. We just say we don’t know why because we don’t eat salty and oily foods. We don’t tell them about the other medication that we are taking.’ (FG1, F, Sesotho)

‘It is not easy to report to the nurse that you are taking something else.’ (FG2, F, Sesotho)

### Theme 5: Healthcare providers do not have time to engage

Respondents mentioned that HCPs do not have time to explain or respond to their questions regarding their conditions or treatment:

‘The nurses, normally ask, what seems to be the problem today, they will then write notes on the file and direct you to where your medication will be dispensed.’ (FG3, F, isiZulu)

‘They don’t have time to answer all our questions because of the queue. Instead of explaining they tell us about queues.’ (FG1, F, Sesotho)

‘The doctors are always in a hurry they don’t want to hear any complaints, especially during December.’ (FG2, F, Sesotho)

‘They do not explain things at all.’ (FG3, F, Sesotho)

#### Sub-theme 3: Nurses’ attitude

Respondents described the nurses’ unpleasant behaviours towards them as being unapproachable, impatient and getting easily annoyed with them:

‘The nurses are not easily approachable.’ (FG2, F, Sesotho)

‘They must understand that nursing is a calling. It is not for them to be impatient with us.’ (FG3, F, isiZulu)

‘Even when you humble yourself and explain how you are feeling. They just looked at you.’ (FG3, F, isiZulu)

‘They get annoyed a lot. I was in the hospital, there was a man lying down in pain. When the nurse came in, she passed a silly remark that she is annoyed already by the man lying down.’ (FG1, F, Sesotho)

‘With a nurse, when you ask, they will say you ask a lot of questions, and they don’t have time.’ (FG2, F, Sesotho)

## Discussion

The themes and subthemes identified in this study offer a comprehensive description of factors that influence the use of HM among patients visiting PHC clinics.

The study revealed that the driving force behind the use of HM is easy accessibility, lower cost and the perception of having no side effects. The findings also found that respondents follow what their forefathers have been doing by using HM. These findings are similar to studies conducted in South Africa^38,39^ and support the notion that HM plays a role in the lives of the population of South Africa.

Because of the lack of knowledge and personal perceptions, respondents raised their concerns about the side effects they are experiencing while using prescribed medicine.^40,41,42^ As part of the consultation and the right of the patient to be informed, HCPs need to discuss the benefits and risks of the prescribed medication with patients.^43,44^ In order to better understand HM and HDIs, an expansion of knowledge of pharmacodynamics and pharmacokinetics of HM is essential. Patients need to hear about HM directly from HCPs to limit HDIs.^45^

Respondents reported that they started using HM upon recommendation by people who have personal experience with HM and television advertisements. This finding indicates that HM in the community and society at large is an important issue as people are sharing information about medicinal plants or herbs among themselves. This can be attributed to the historical African culture of sharing information about medicinal herbs orally from generation to generation.^46^ Moreover, various media advertisements have increased awareness and have given HM respect and credibility.^10,39,41,47^

During the discussion, most FG respondents confirmed the co-administration of HM and prescribed medication. Concurrent use of these modalities is a common practice, especially in patients with chronic conditions.^23^ This is because of patients simultaneously seeking treatment from both conventional and traditional or complementary health systems for the same ailment.^48^

Even though a small fraction of respondents felt that using HM and prescribed medicine is wrong, they took this decision because of how they felt, not necessarily because they had the knowledge regarding HDI. This highlights the importance of educating patients about the danger of co-administering HM and prescribed medicine and possible HDIs.^23,49,50^

Herbal medicines contain hundreds of active ingredients that may interact and cause modification of prescribed medication.^48^ This study revealed a low awareness of potential herb-drug interaction where most of the respondents mentioned that they were not aware of the potential interaction of herbs and prescribed medication. A similar study carried out by Ezuruike and Prieto^51^ showed that 60% of participants who took HM alongside prescribed medicine are unaware of the identity of HM being taken, which exacerbates the risks of the HDIs. A study conducted in South Africa showed similar results where consumers purchase HM products from herbalists and traditional healers, packaged in containers with the contents not being stated.^20^ These findings demonstrate that patients do not have knowledge of HDIs, and there is a need for continuous education of patients about HDIs.

Respondents in this study mentioned that they do not disclose the use of HM to their HCPs. This shows that patients are not forthcoming with their use of HM when consulting in the PHC clinics. A possible reason could be the perceived unpleasant behaviours of PHC nurses mentioned in the study.^27,31^ Respondents confirmed that they experience unpleasant behaviours from nurses during clinic visits. This behaviour of PHC nurses could weaken the ability of the health system and compromise the quality of patient care.

Respondents also mentioned that doctors and nurses do not have time to give an explanation about their conditions or treatment during the consultation. This may be because of a few HCPs having to deal with the volume of patients in a short time.^52^ A study conducted in a hospital in South Africa reported that nurses experience a high patient load, staff absenteeism and burnout.^53^ This calls for stakeholders to come up with interventions to combat workload and absenteeism to create favourable conditions for both patients and HCPs. Education about the use of HM requires a sensitive approach that depends on listening and spending time with the patients.^45^

### Strength and limitations

This study provided insight and understanding into the use of HM by patients attending PHC clinics. The study also encourages PHC providers to communicate and inform patients about possible herb-drug interactions.

The limitation of this study is that the research was conducted in only three provinces; therefore, the findings cannot represent the wider views of the population of South Africa attending PHC clinics. Another limitation is that the study did not recruit participants from all ethnic groups. Despite these limitations, the findings of the study can be transferable to similar settings in other provinces.

### Recommendations

The lack of enquiry and monitoring of the co-administration of prescribed and herbal medicines on patients is a significant patient safety risk. To ensure that patients are aware of herb-drug interaction, PHC stakeholders must make it mandatory that communication about the use of HM is part of the consultation. Additionally, more research needs to contribute to the awareness and education of patients about HDI.

## Conclusion

The findings of this study revealed that there is a lack of knowledge and awareness of the risks that may be with the co-administration of HM and prescribed medicines. Within the identified themes, participants believed anecdotes from community members and family, cultural background and positive experiences of HM. These findings can be used to develop interventions aimed at preventing possible HDIs.
